# Virus-host interactions in iron-oxidizing mats of the East Pacific Rise

**DOI:** 10.1371/journal.pone.0357099

**Published:** 2026-09-08

**Authors:** Kathryn L. Campbell, Jason B. Sylvan, Craig L. Moyer, Heather Fullerton, Jessica M. Labonté

**Affiliations:** 1 Department of Marine Biology, Texas A&M University at Galveston, Galveston, Texas, United States of America; 2 Department of Evolution, Ecology, and Organismal Biology, The Ohio State University, Columbus, Ohio, United States of America; 3 Department of Oceanography, Texas A&M University, College Station, Texas, United States of America; 4 Biology Department, Bellingham, Washington, United States of America; 5 College of Charleston, Charleston, South Carolina, United States of America; Friedrich Schiller University, GERMANY

## Abstract

Iron-oxidizing microbial mats are commonly encountered features at low-temperature (<100˚C) hydrothermal vents found along the seafloor at tectonically active settings. The microbial communities in these mats are uniquely adapted to exploit the specific ecological niches within the physical and chemical gradients of their environment. A vital component for these adaptations is viral infections, although their impacts are poorly constrained. While the microbial communities of iron-oxidizing mats at seafloor hydrothermal vents have been well studied, the role of viruses in affecting these communities with respect to biogeochemical cycling, diversity, and/or population control remains unclear. The goal of this study was to assess the role and impact of viruses within iron-oxidizing mats of the well-studied 9˚N East Pacific Rise (EPR) segment. We found unique viral assemblages at each site, with 41% of the viral operational taxonomic units (vOTUs) shared across all sites, but with differing relative abundances. The virally-encoded auxiliary metabolic genes (AMGs) were also differentially abundant among the sites and included processes related to carbon and sulfur cycling. Virus–host linkages showed that the viral assemblages infect different members of the microbial communities within each mat, as well as the potential to affect ecological shifts in the metabolisms of various hosts due to viral infection, which could affect the rates of microbial processes. In these iron-oxidizing mats, viruses are therefore likely playing a role in the diversity and evolution of the microbial taxa through lysis and the potential to alter host metabolisms. Exploration of virus–host dynamics within iron-oxidizing mats in hydrothermal vent systems like the EPR aid in constraining a vital component of the deep ocean food webs and give critical insight to the ecology of these productive microbial communities.

## Introduction

Hydrothermal vent habitats support abundant and diverse chemosynthetic microbial communities and their associated food webs. While the microbial assemblages have been studied since the discovery of hydrothermal venting [1–4], the role viruses play within these environments is largely unknown. Viruses are pivotal components of microbial communities as they are catalysts for microbial adaptation to changing environmental conditions [5–7], a significant source of microbial mortality through lysis [5,8], and viruses affect biogeochemical cycles [9,10]. Understanding the role viruses play within hydrothermal vent microbial communities is critical to understanding community dynamics that could affect primary productivity [1] and consequently secondary production [2,11].

One common feature of hydrothermal vent habitats is diffuse flow vents, which form when warm subsurface fluids mix in the shallow subseafloor with oxygenated bottom water, resulting in warm (<100˚C), diffusely flowing fluids exiting the seafloor. This creates habitats for chemolithoautotrophic organisms that are hot spots of microbial activity and are often associated with iron-oxidizing microbial mats [3]. Zetaproteobacteria are key microbes that use the diffuse Fe(II)-rich fluids to engineer the mat habitat via neutrophilic iron oxidation, which results in deposition of iron-oxide stalks [12–16]. Zetaproteobacteria serve as the most abundant primary producers in these iron-oxide microbial mats and are supported by a variety of microbial members capable of iron reduction, carbon fixation, fermentation, nitrate assimilation, and denitrification, among others [17–19]. Heterotrophs and fermenters are provided a reservoir of organic carbon from the polysaccharides and adsorbed organic carbon contained in the iron-oxyhydroxide stalk structures produced through Zetaproteobacteria metabolism [19–21]. It is unclear how the viruses of these prokaryotic communities, while present [19], affect biogeochemical cycles, diversity, and/or exert population control within the food web of these iron mats. Diversity, high biomass, and metabolic cooperation that are common in iron-oxidizing mats [19] can result in unique virus–host interactions.

Phages, viruses that infect bacteria, can have a range of interactions with their host that encompass both antagonistic and/or mutualistic relationships that alter and affect the diversity of microbial populations and contribute to biogeochemical processes [22]. Lytic viruses (lytic cycle: commandeer host, replicate, and lyse host) can redirect the metabolic activity of their hosts as well as release labile nutrients through lysis, providing food for the surrounding microbial community and altering biogeochemical cycles; an overall process known as the viral shunt [23,24]. In contrast, temperate phages (lysogenic cycle: integrates into host genome and passively replicates with host cell or enters lytic cycle) have a relationship with their host that is mutualistic prior to entering the lytic phase; it can lead to lysogenic conversion, where phage integration into the host genome results in a new stable phenotype or phenotypic plasticity [6]. Lysogenic conversion can increase fitness by allowing hosts to occupy novel niches within the environment, increasing the survivability of the host and phage simultaneously [6]. High incidences of temperate phages have been found in diffuse flow fluids [25,26], which may result from the diverse and unpredictable conditions associated with diffuse flow vents making it challenging for free-living organisms and therefore selecting for a survival strategy that is more mutualistic in nature. A shared component between these two types of infection cycles are phage encoded auxiliary metabolic genes (AMGs), which allow phages to metabolically reprogram their host during infection, affecting diversity and function of marine microbial populations [5]. Phages encode genes that participate in novel pathways in hydrothermal sediments [10] and plumes [9], allowing them to directly participate in important biogeochemical cycles.

Given the integral role that viruses play within microbial communities in general, it is likely that there are important host–virus interactions within iron-oxidizing mats that help shape and sustain these isolated environments as well as influence important ecological processes in these mats, such as iron oxidation, carbon cycling, and nitrate reduction. To date, viral ecology of iron-oxidizing mats in hydrothermal vent habitats is unexplored. Therefore, the goal of this work was to provide a first assessment **of** the role and impact of viruses in iron-oxidizing microbial mats. We chose the well-studied 9º50’ N segment of the East Pacific Rise as a field site and we hypothesized that the viral community would reflect key iron-oxidizing microbial mat community members as well as encode AMGs that are ecologically important for the various microbial metabolic processes (e.g., iron, sulfur, carbon, and nitrogen cycling). We sampled three iron-oxidizing mats, induced and collected the viral communities, and present the analysis of their metagenomes here.

## Materials and methods

### Sampling

**Fig 1:**
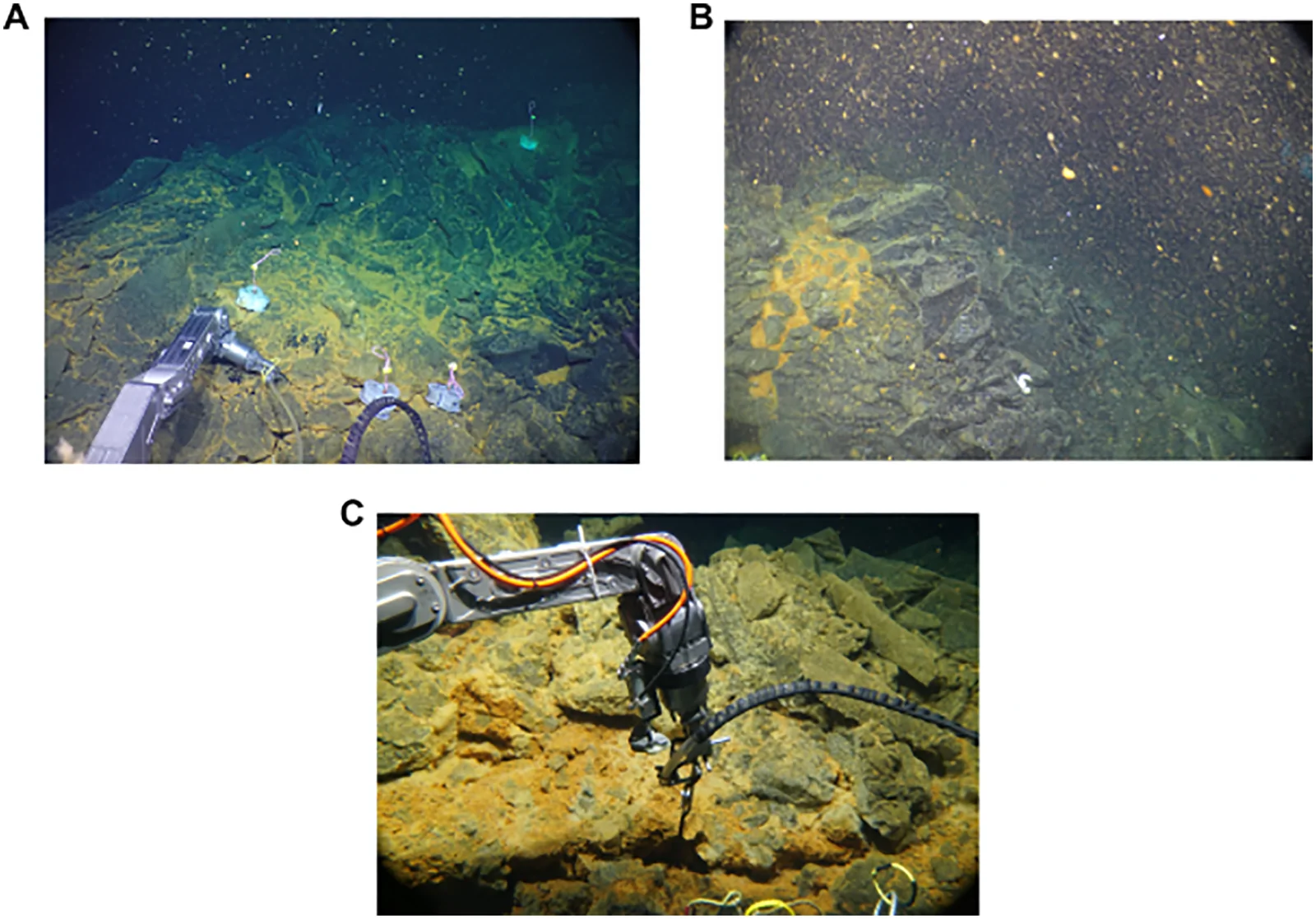
Images from sampling sites taken aboard HOV Alvin. A) Marker 28 location (dive 5020). B) Io location (dive 5022). C) Riftia Field location (dive 5024). Reproduced under a CC BY license, with permission from Woods Hole Oceanographic Institution (WHOI), original copyright 2020.

In collaboration with the hot2cold Vents project [1,27], samples were taken during cruise AT42–09 (March/April of 2019) aboard the *R/V Atlantis* at 9°50’N segment of the East Pacific Rise, which is a fast-spreading mid-ocean ridge, with a spreading rate of ~5.5 cm yr^-1^ [28]. Three flocculent iron-oxidizing mats found in diffuse flow areas, characteristically brown/orange in color, were collected using a hydraulic slurp sampler aboard HOV Alvin on dives 5020 (Marker 28), 5022 (Io) and 5024 (Riftia field −1, 2) (Fig 1, Table 1). The hydraulic slurp sampler was used here in single chamber mode – a water pump applies vacuum via a hose connected to one liter chamber (bottle) and collects material sucked up as the hose is applied to a surface (microbial mats in this case). A mesh screen at the top of the chamber filters out rocks and macrofauna. The collected sample remains in the chamber on the basked of HOV Alvin until recovery after the HOV is on deck. Temperatures of hydrothermal fluids exiting the seafloor closest to the collected mats were measured using the resistance temperature device (RTD) aboard HOV Alvin. Additional background seawater was also collected using a 1.2 L Niskin bottle connected to HOV Alvin. Once onboard the ship, the mat samples were subsampled from the slurp chambers into two sets of subsamples: (1) microbial mat collected into 50 ml centrifuge tubes and stored at 4°C until processing for viral induction experiments 14 months later, and (2) microbial mat mixed with an equal volume of RNALater and stored for 24 hours at 4ºC before being frozen at −80ºC until further processing to determine initial bacterial community composition. The Riftia Field samples are subsets of the same sampled mat.

**Table 1:** Sample recovery of iron-oxidizing mats.

Sample IDs	Sample Dive	Sample Site	Sample Coordinates	Depth (m)	Volume (mL)	Temperature (°C)
M28	5020	Marker 28	9.83614˚N, 104.29089W	2509	20	12.5
IO	5022	Io	9.83512˚N, 104.29100˚W	2507	27	10
RF1	5024−1	Riftia Field	9.84548˚N, 104.29300˚W	2510	27.5	16
RF2	5024−2	Riftia Field	9.84548˚N, 104.29300˚W	2510	30	16

### Mitomycin C prophage induction of microbial mats

The permeate, or flow-through, of tangential flow filtration (TFF) [30 kDa TFF PrepScale (MilliPore)] of background seawater was used to generate virus-free seawater shipboard and kept in the dark until use. After a 14-month storage period, enough virus-free seawater was added to bring the volume up to 50 ml for each sample. The samples were gently mixed using sterile serological pipettes, then split into equal volumes in two sterile 50 ml centrifuge tubes, one for the control (i.e., uninduced) and one for the induction experiment. For each of the induction samples, mitomycin C was added to a final concentration of 1 μg/ml and inverted to mix [29]. All samples were incubated at 13°C for 24 hours. After the incubation period the samples were spun at 4,000 x g for 45 minutes and the supernatants were filtered through a 0.2 μm syringe filter (Supor® membrane, Pall Laboratory) into sterile 50 ml centrifuge tubes. The filtrate was stored at 4°C until DNA extraction, which was less than a week after the incubations were completed.

### DNA extraction and sequencing

Samples were concentrated using Sartorius VivaSpin Turbo 15 columns with a 30,000 MWCO filter to a final volume of <1 ml. Each sample was washed 3x in the columns with 15 ml virus-free seawater and eluted to a final volume between 1.5-1.75 ml. DNA was extracted using Qiagen DNeasy PowerSoil kit (Qiagen, MD) for DNA using the manufacturer’s protocol. Due to low DNA recovery, samples were subjected to whole genome amplification (WGA) with Qiagen Repli-g Mini kit (Qiagen, MD). Each sample (controls and induced for all four samples) was amplified in triplicate using 5 µl of template per reaction tube and following the manufacturer’s protocol. The samples were then cleaned using AMPure XL beads (Beckman, CA) according to the manufacturer’s recommendations. All samples (0.018–3 μg) were sent for library preparation (PerkinElmer NEXTFLEX Rapid XP DNA-Seq Kit HT) and sequencing (32M reads per sample) to TAMU AgriLife Genomics and Bioinformatics Service using Illumina NovaSeq with 150 bp paired-ends.

To determine initial bacterial community composition, DNA was extracted as previously described [30]. Briefly, DNA was extracted from 0.5g of microbial mat material using the manufacturer’s protocol with minor modifications (FastDNA SPIN Kit for Soil, MP Biomedicals, Santa Ana, CA). In place of the sodium phosphate buffer, 250µl of 0.5M sodium citrate, pH 5.8 was used, and lysis was performed using two rounds of bead beating at a setting of 5.5 using the FastPrep instrument, with samples being placed on ice between rounds (MP Biomedicals). Then quantified by a Qubit 4.0 fluorometer with high-sensitivity reagents (ThermoFisher Scientific, Waltham, MA). The bacterial V3-V4 SSU rRNA gene was amplified by PCR using 340F and 784R primers with Illumina adaptors [30,31] by NC State Genomic Sciences Laboratory. The resulting amplicons were sequenced using a MiSeq (Illumina, San Diego, CA) following the manufacturer’s protocol to generate 2 X 300 bp reads. After sequencing, reads were trimmed of primers using CutAdapt (v 3.5) [32]. Amplicon sequence data processing and analysis were performed using a pipeline involving the DADA2 (v 1.34.0) [33] and Phyloseq [34] packages in R (version 4.4.0). ASVs were created using default commands in DADA2, followed by removing Chimeric sequences using the removeBimeraDenovo function and the consensus method. The SILVA SSU rRNA database (v 138.1) was used for the taxonomic classification of ASVs. DNA was extracted only from Marker 28 and Riftia Fields due to constraints imposed by field sampling.

### Viral metagenome analysis

Sequences were quality controlled using BBtools to trim adapter sequences (bbduk) using ‘ktrim=r k=23 mink=11 hdist=1 qtrim=r trimq=20 tpe tbo’, merge pair-end reads (bbmerge) with parameters ‘rem k=62 extend2=50 ecct vstrict’, remove contamination (bbduk) with parameters ‘minid=0.95 maxindel=3 bwr=0.16 bw=12 qtrim=rl trimq=10 untrim’ and mask low-complexity regions (bbmask) with ‘entropy=0.50’ [35]. Sequences were assembled using MEGAHIT v1.2.8 [36] with default parameters. Viral identification was performed on the individual assemblies with VIBRANT (v1.2.1) [37] using the ‘-virome’ flag. The resulting viral sequences were then filtered by length to keep viral contigs ≥ 5 kbp before clustering into viral operational taxonomic units (vOTUs) using CD-HIT-EST v4.8.1 [38] at 95% ANI and 85% alignment coverage. A cut off of 5 kb (instead of 10 kb) was used to account for the genomes of single stranded DNA (ssDNA) viruses, which can be between 5.3–6.3 kb, given the likelihood of ssDNA virus overamplification during WGA [39–41]. The quality-controlled reads (from BBTools) were mapped back to the vOTUs using bwa-mem2 v2.2.1 [42] and the number of mapped reads to each vOTU was calculated using samtools [43]. Taxonomy of each of the vOTUs was performed using geNomad v1.5.2 [44] and any unclassified vOTUs were categorized as “Unknown viruses”. AMGs identifications were compiled from the VIBRANT output for each vOTU. Lifestyles of vOTUs were ignored due to the inconsistencies and variability associated with mitomycin C inductions in terms of producing reliable chemical induction of lysogens; additionally, the use of WGA would potentially skew any lifestyles predicted and enumerated via relative abundance and therefore would be unreliable distinctions [45,46]. Hosts of the vOTUs were predicted utilizing iPHoP v1.3.3 [47], which uses both host-based (tRNAs, CRISPRs, etc) and phage-based tools (machine-learning approach based on association of viral genes to hosts) [48–51]. The resulting host predictions were additionally filtered to only keep the ones that were either called by more than one tool or had ≥ 95% confidence score. The resulting hosts were compared to amplicon sequence variants of the 16S rRNA gene from the bulk mat for Marker 28 and Riftia Field sites. Tabular results for each of the various software used can be found in S2-5 Tables in S1 File.

### Statistical analysis

To assess the differences between the viral populations, a Bray-Curtis dissimilarity matrix was calculated with the vegan package [52] in R [53] using the number of reads mapped per vOTU per viral metagenome $\frac{\frac{Mapped\;reads}{Size\;of\;vOTU\;genome\;(kbp)}}{Size\;of\;whole\;metagenome\;(Mbp)}$

as count data for each sample (n = 8 samples). From this matrix, a principal coordinate analysis was performed using the ape package [54] in R. To assess the statistical significance of the differences associated with the PCoA, a PERMANOVA was performed using the vegan package [52]. To directly compare the abundances of vOTUs across sites, the z-score was calculated by vOTU to convert values to standard deviations $\left[Z=\;\frac{abundance-mean\;abundance}{standard\;deviation\;of\;abundance}\right]$. A heatmap of the z-score values of all vOTUs was generated using ggplot2 in R [55]. To compare the AMGs between sites, a dissimilarity matrix was calculated between the AMGs encoded by the vOTUs using the count data associated with each vOTU. The dissimilarity matrix was used to generate a nonmetric multidimensional scaling (NMDS) plot using ggplot2 [55] in R. A PERMANOVA was performed to assess the statistical significance of any differences in the distribution of AMGs by site using the vegan package [52]. AMGs were also compared using z-scores calculated across AMGs, to compare abundances across the sites and visualized using a heatmap using ggplot2 in R [55].

## Results

### Viral assemblage composition

A total of 27,493 viral contigs ≥ 5 kb were assembled. Clustering at 95% ANI resulted in a total of 3,639 viral operational taxonomic units (vOTUs) (Table 2), which were used to assess taxonomy, identify auxiliary metabolic genes (AMGs), and predict potential hosts.

**Fig 2:**
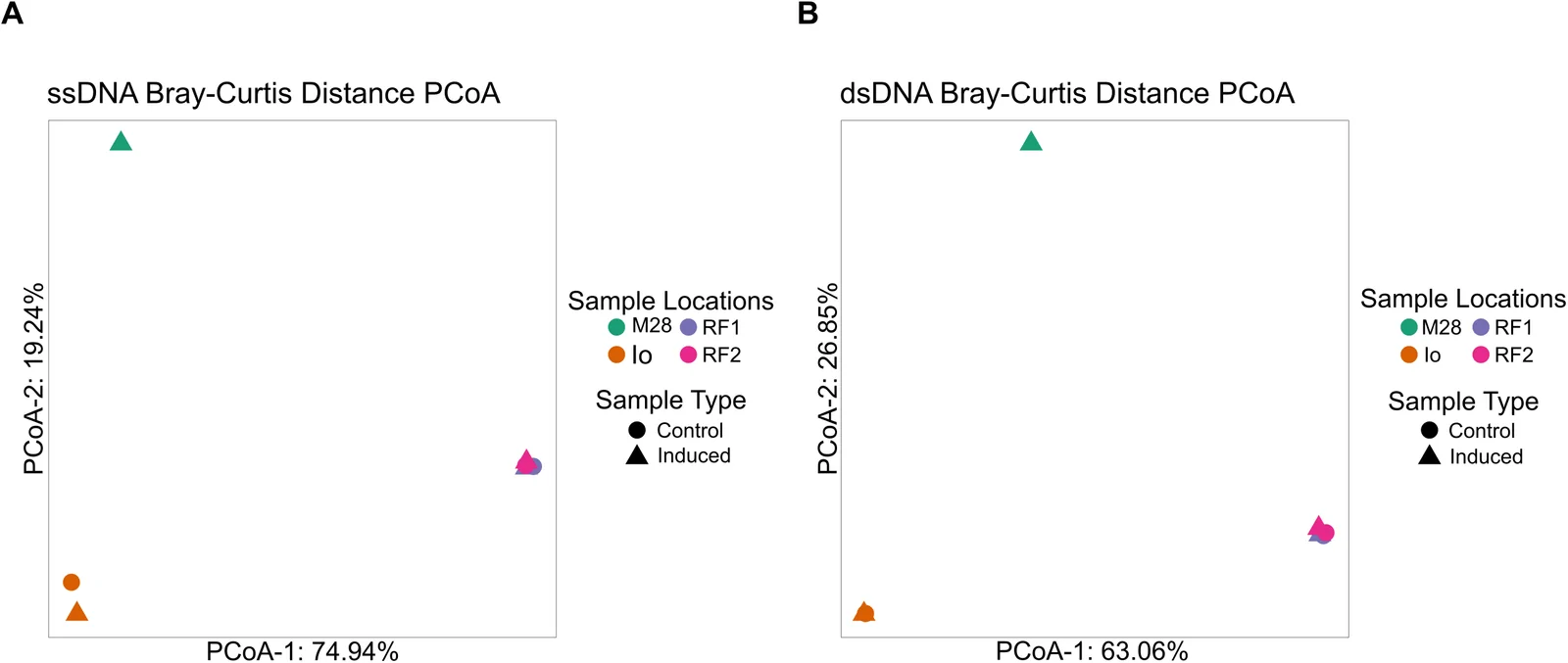
Principal coordinate analysis of vOTUs. Principal coordinate analysis of the Bray-Curtis dissimilarity matrix based on normalized read counts per vOTU (mapped reads per vOTU genome size in kbp per total metagenome size in Mbp) between each site for ssDNA [A] and dsDNA [B] viral populations, which accounts for 94.2% and 92.9% of the variation, respectively. The sites are clustering by location.

Comparison of the viral assemblages between the three sites, Marker 28, Io, and Riftia Field, showed that both the single-stranded DNA (ssDNA) (Fig 2A) and double-stranded DNA (dsDNA) (Fig 2B) viral populations were distinct, which were statistically significant (*p-value < 0.05*).

**Table 2:** Summary of the reads and vOTUs for each sampling location.

	Marker 28	Io		Riftia Field			
Sample	Induced	Control	Induced	Control	Induced	Control	Induced
Total # reads (x10^6^)	2.9	32.9	50.0	55.7	44.7	47.5	49.0
#vOTUs	2143	2518	2388	3085	3098	2796	2821

**Fig 3:**
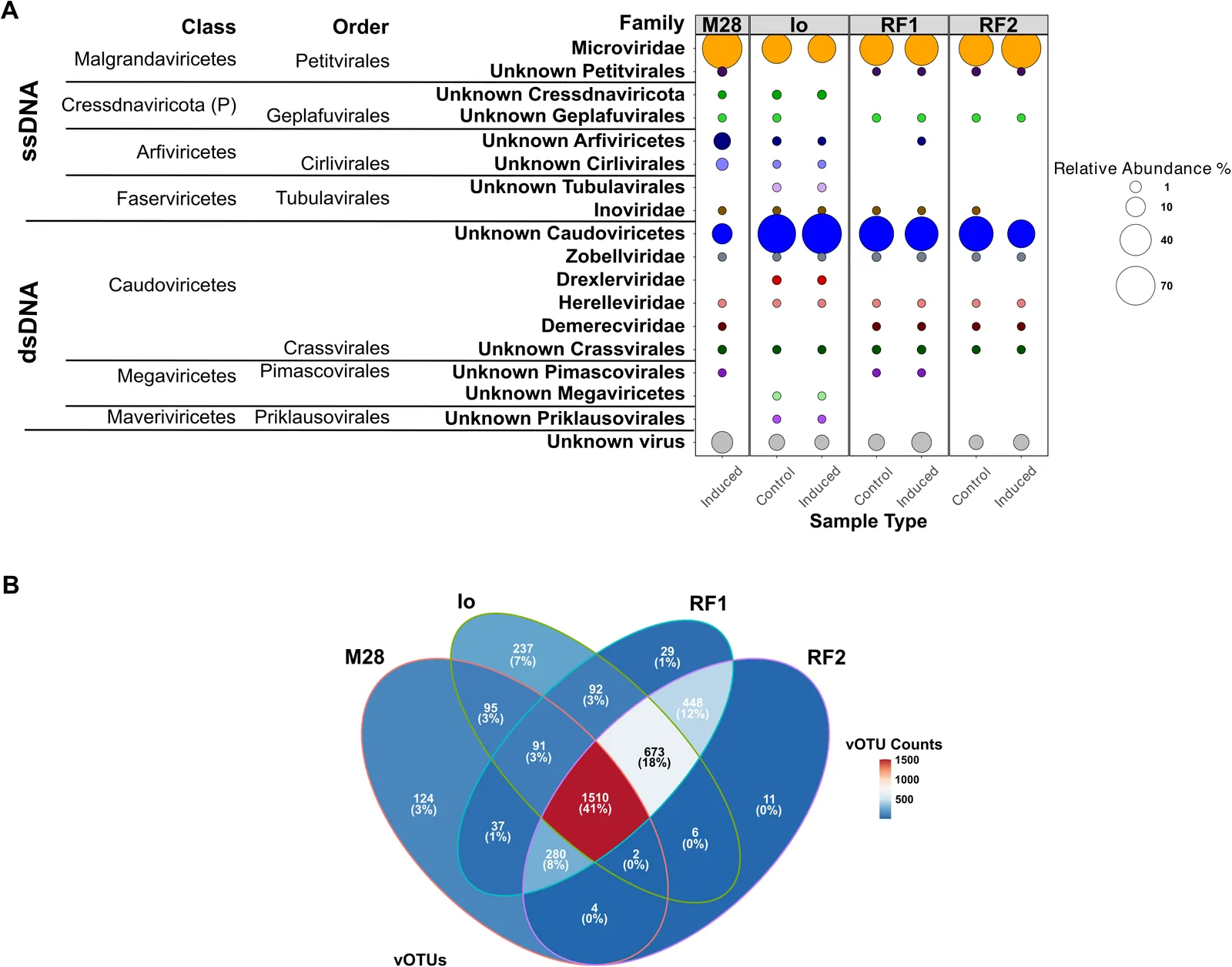
Taxonomy and relative abundance of the viral assemblages from each site (A) and Venn diagrams of the distribution of the viruses across the different sites using counts of vOTUs (B). The assemblages were dominated by *Microviridae*, unknown viruses of class Caudoviricetes, and Unknown viruses (i.e., unclassified viruses). 41% of the vOTUs were shared between the three sites.

Most of the viruses across all samples occupied either the designation of an unknown virus (2.6-24.1%), unknown class Caudoviricetes (10.2-75.5%), or family *Microviridae* (0.08-70.9%) (Fig 3A). The remainder of the composition varied and consisted of no more than ~5% of the relative abundance. The majority were classified within ssDNA viruses and dsDNA. ssDNA viruses that were shared across all sites included members of the family *Inoviridae*, and viruses from orders Geplafuvirales, and class Arfiviricetes. Marker 28 and Io sites shared viruses from class Cressdnaviricota and order Cirlivirales. Marker 28 and both Riftia Field samples shared viruses from order Petitvirales. dsDNA viruses that were shared across all sample sites from class Caudoviricetes included *Zobellviridae*, *Herelleviridae*, and viruses from order Crassvirales. *Drexlerviridae* was only found at Io sites, while *Demericviridae* was shared between Marker 28 and both Riftia Field samples. Giant viruses of the order Pimascovirales were observed at Marker 28 and one of the Riftia Field samples. Site Io also was observed to include the presence of giant viruses, but from class Megaviricetes and virophages from order Priklausovirales that were unique to the Io site.

**Fig 4:**
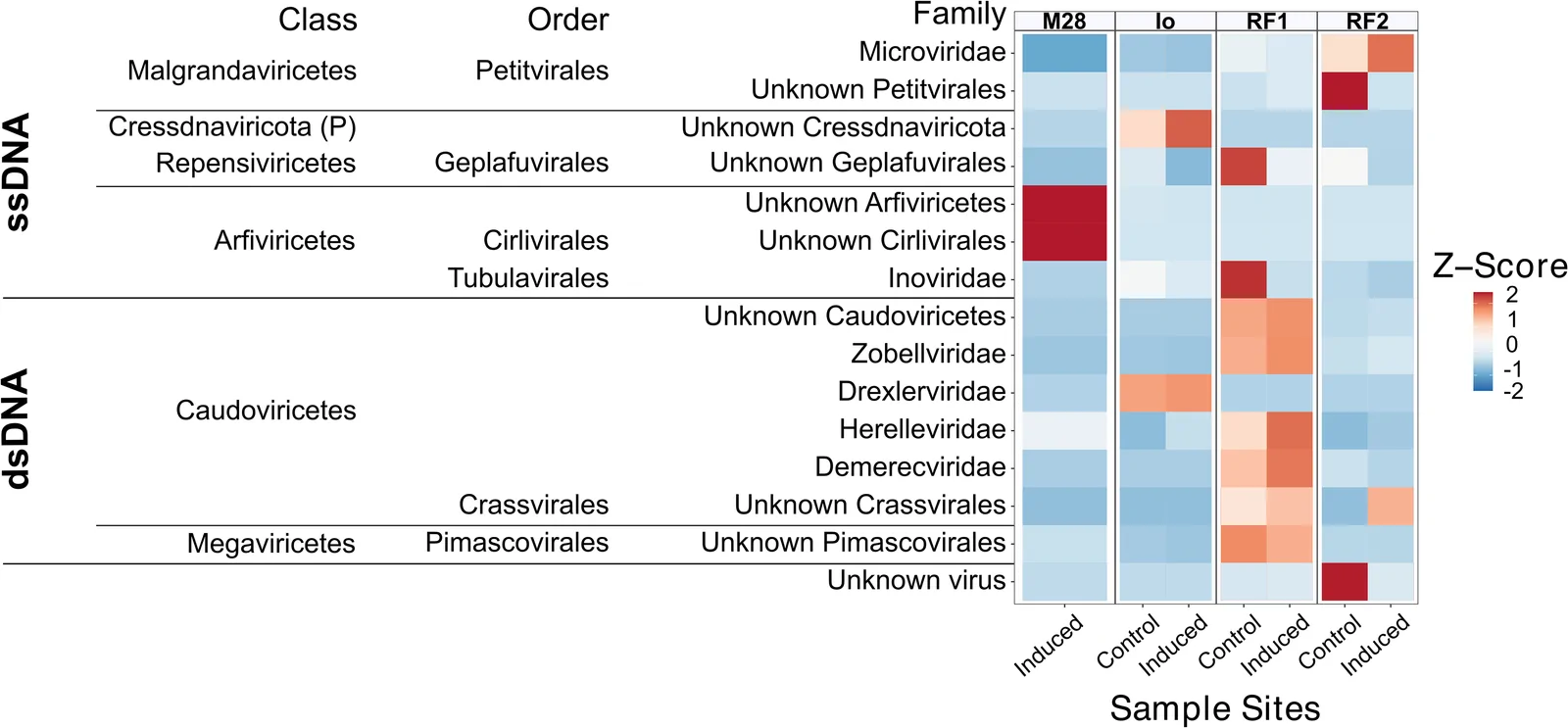
Heatmap of the z-score of the vOTU population abundance across each sampling site. The viral assemblages at each site, while sharing 41% of all viruses, differ in their population abundance. Red indicates population abundances above the mean, while blue indicates population abundances below the mean in units of standard deviations.

The viral community compositions were distinct between each of the sites (Fig 2), although the vOTUs unique to each site accounted for less than 7% of all vOTUs. Marker 28, Io, and the two Riftia Field samples harbored 124 unique vOTUs (3% of all vOTUs), 237 (7%), 29 (1%) and 11 (<1%), respectively (Fig 3B). Despite 41% of the vOTUs being shared between the sites (Fig 3B), the vOTUs exhibit variable abundances where each site has a unique assemblage of vOTUs (Fig 4).

### Viral AMG metabolisms and pathways

**Fig 5:**
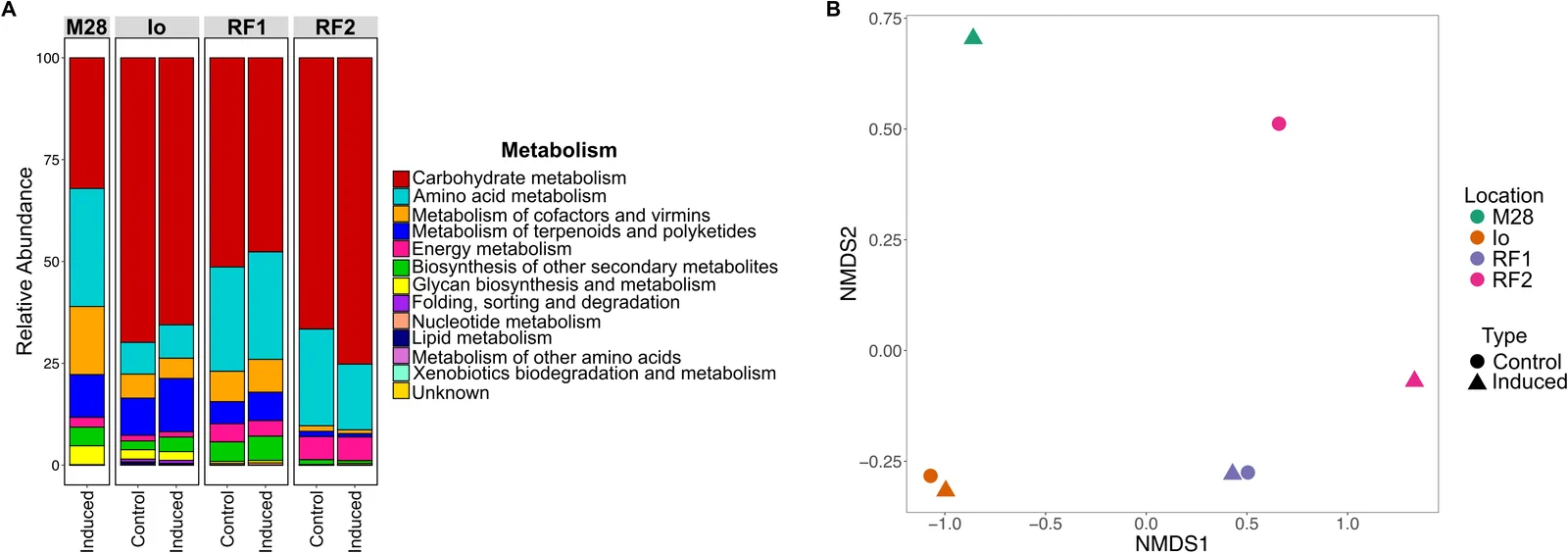
Comparison of virally-encoded genes. A) Relative abundances of virally-encoded metabolisms. Stacked bar histogram of the various metabolisms present within the vOTUs. The relative abundance of each AMG is commensurate with the relative abundance of the associated vOTU. B) Bray-Curtis NMDS of AMGs. The distribution of AMGs at each of the sample sites were compared using a Bray-Curtis dissimilarity matrix. As observed with the viral composition comparison, each site groups separately.

There was a total of 72,783 proteins found in the vOTUs, of which only 4,904 (6.7%) were annotated. AMGs were identified in 10.7% of the vOTUs, representing a total of 557 proteins (0.77%). The function of these proteins consisted of carbohydrate metabolisms, amino acid metabolism, metabolism of cofactors and vitamins, metabolism of terpenoids and polyketides, energy metabolisms, biosynthesis of other secondary metabolisms, and glycan biosynthesis and metabolism (Fig 5A). Despite these similarities, the AMGs were distinct among each site (Fig 5B), which may be a result of the differences in relative abundances of the vOTUs and the AMG composition of each site. However, the differences were determined not to be statistically significant (*p-value>0.05*).

**Fig 6:**
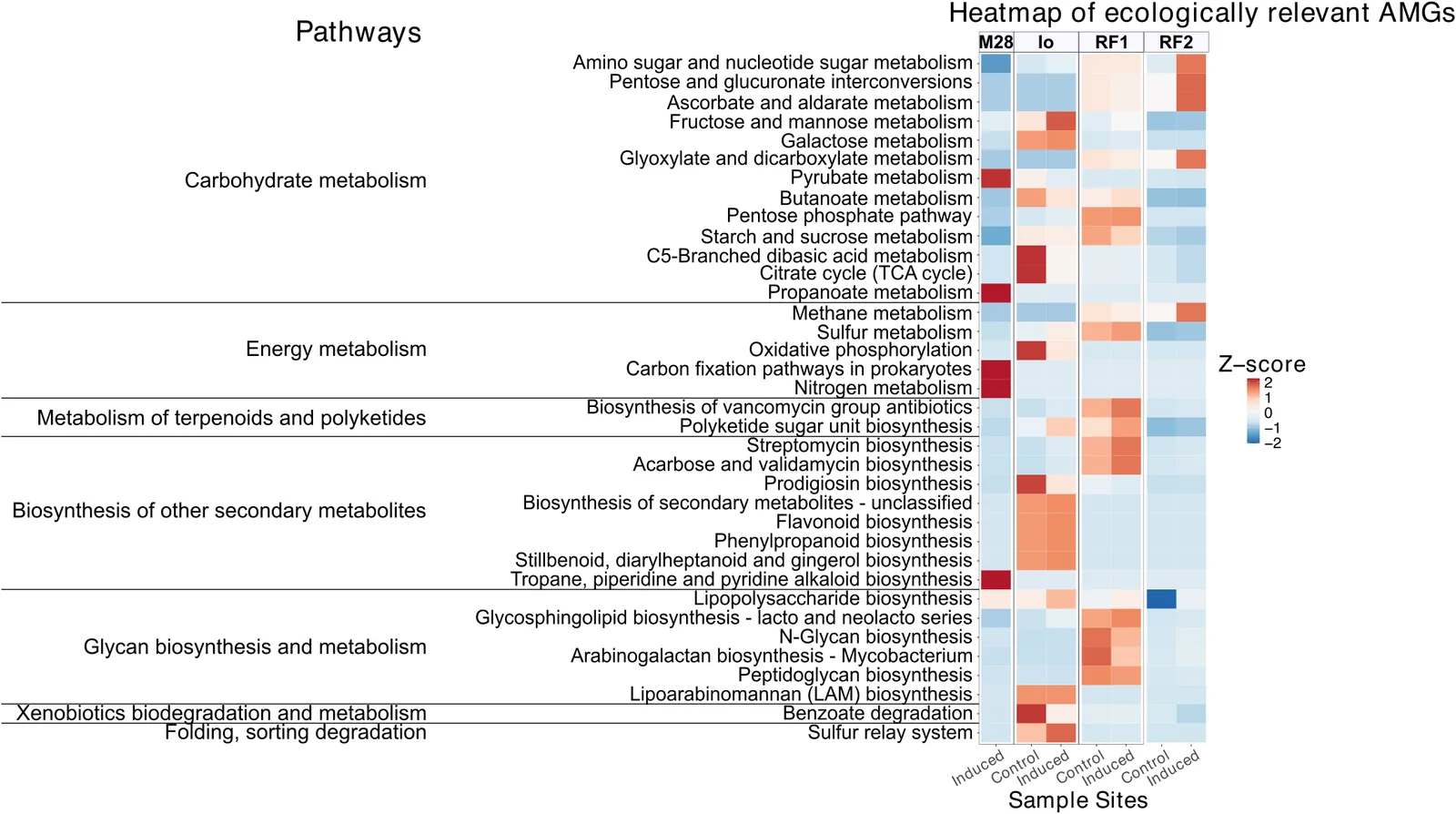
Heatmap of the different ecologically relevant metabolisms. The z-score was calculated using relative abundance across each of the AMGs to compare between the sites. Red indicates relative abundances above the mean, while blue indicates relative abundances below the mean in units of standard deviations.

In order to understand the potential ecological impacts of viruses in iron-oxidizing mats, we focused on the specific processes that affect the physiology of the hosts. These processes include carbohydrate metabolisms, energy metabolisms, biosynthesis of other secondary metabolites, metabolism of terpenoids and polyketides, glycan biosynthesis and metabolism, as well as folding, sorting and degradation within the sulfur relay system, and xenobiotics biodegradation and metabolism. Each sampling site showed different relative abundances of the various pathways (Fig 6), although the differences were not statistically significant (*p-value>0.05*).

For carbohydrate metabolisms, Marker 28 site had relative abundances above the mean compared to the other sites for AMGs classified for propanoate metabolism and pyruvate metabolism; Io site had relative abundances above the mean in AMGs for fructose and mannose metabolism and galactose metabolism as well as for butanoate metabolism. The Riftia Field site showed the most carbohydrate pathways including amino sugar and nucleotide sugar metabolism, pentose and glucuronate interconversions, ascorbate and aldarate metabolism, and glyoxylate and dicarboxylate metabolism present above the mean for both samples and butanoate metabolism, pentose phosphate pathway, and starch and sucrose metabolism present at Riftia Field sample 1 but not sample 2 (Fig 6).

Relative abundances of AMGs related to energy metabolisms and carbon fixation were also different among the sites. Genes for methane metabolism were present at the greatest abundance compared to the mean in both Riftia Field samples, while AMGs for sulfur metabolism were only elevated above the mean in Riftia Field sample 1. Marker 28 showed higher than the mean relative abundance for carbon fixation pathways and nitrogen metabolism for all sites. vOTUs from Io had the highest relative abundance of oxidative phosphorylation AMGs and some connected to sulfur metabolism.

AMGs for biosynthesis of other secondary metabolites and metabolism of terpenoids and polyketides were higher than the mean for Io and Riftia Field sites. Microbial mats from Io harbored AMGs above the mean relative abundance for polyketide sugar unit biosynthesis, flavonoid biosynthesis, phenylpropanoid biosynthesis, stillbenoid diarylheltanoid, and gingerol biosynthesis and other unclassified secondary metabolites. The Riftia Field sample 1 was above the mean for AMGs related to biosynthesis of vancomycin group of antibiotics, polyketide sugar unit biosynthesis, streptomycin biosynthesis, and acarbose and validamycin biosynthesis. Marker 28 had elevated abundance for tropane, piperidine and pyridine alkaloid biosynthesis.

The relative abundance of AMGs responsible for glycan biosynthesis and metabolism was different for each site as well. Marker 28, Io, and Riftia Field sample 1 showed higher than the mean abundance of AMGs for lipopolysaccharide biosynthesis. Io also showed elevated abundance of lipoarabinomannan biosynthesis. The Riftia Field sample 1 had higher than the mean abundance in AMGs for glycosphingolipid biosynthesis, N-glycan biosynthesis, arabinogalactan biosynthesis, and peptidoglycan biosynthesis. The sample from Io was the one that showed high abundance of AMGs that participate in benzoate degradation and sulfur relay system.

### Predicted hosts

**Fig 7:**
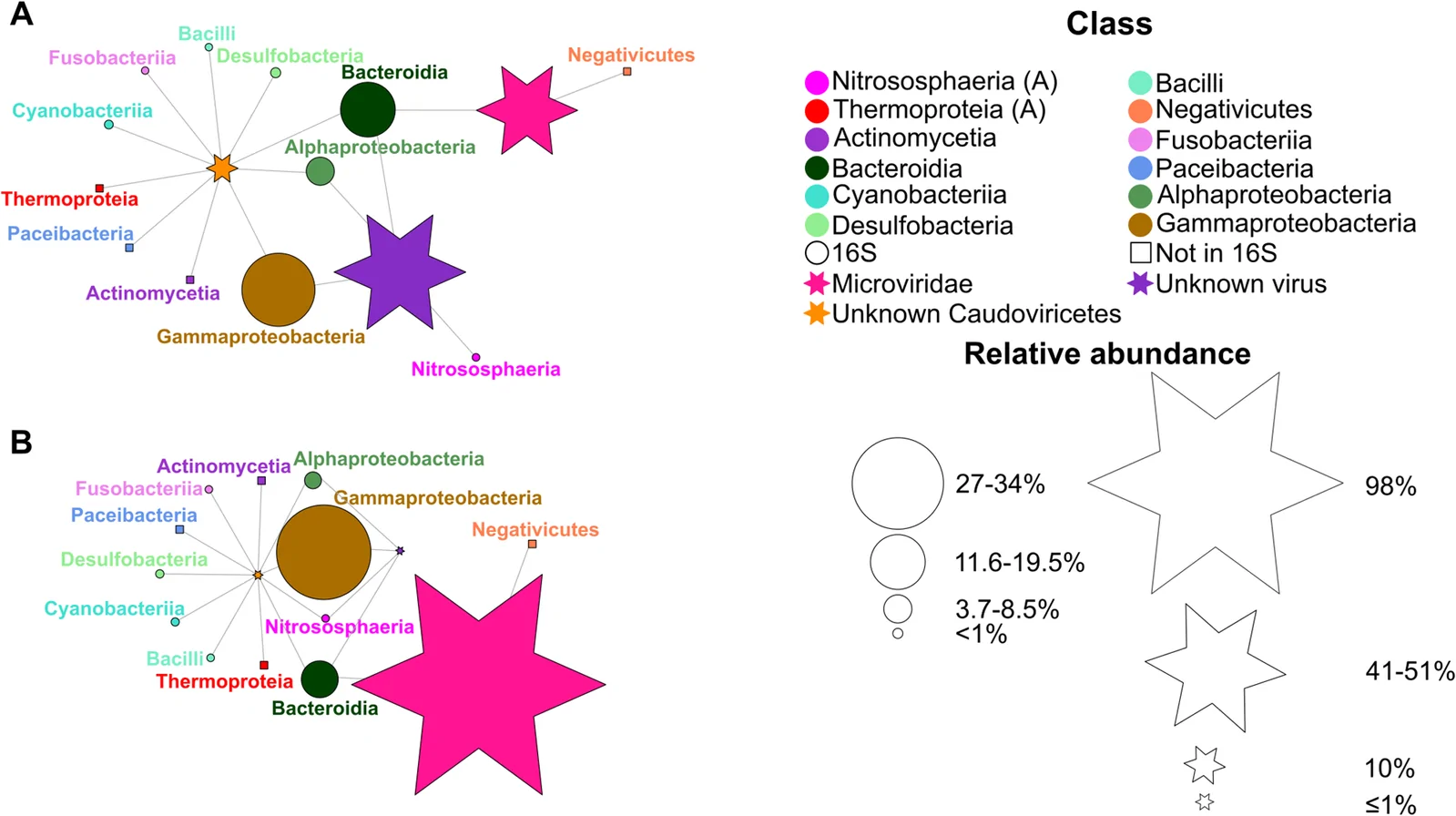
Network of predicted hosts. The predicted hosts and their associated viral families of Marker 28 (A) and an average across both control and induced samples of Riftia Field (B). Size and shape of host class (circles) was determined by its presence within the 16S rRNA gene community. If present, the class was represented by a circle and sized by the relative abundance associated with the 16S rRNA gene community. If the class was not present, the class was represented by a square using the smallest size (i.e., not associated with a relative abundance). The viruses, indicated by the star shape, are also expressed in relative abundance. Prokaryote host classes that contain an (A) are Archaea, ssDNA: Microviridae, dsDNA: Unknown Caudoviricetes, Unknown: unknown virus.

A total of 68 high confidence hosts, encompassing 10 classes of Bacteria and two classes of Archaea, were predicted for the vOTUs, representing ~7.3% of the viral community (Fig 7A, B). Hosts were predicted for *Microviridae* (ssDNA), viruses from class Caudoviricetes (dsDNA), and viruses that were unclassified and reflected prevalent members of the 16S rRNA gene community (S1 Fig) with classes Gammaproteobacteria (27.1-34.6%, including order Methylococcales at 1.4-20.5%), Zetaproteobacteria (9.2-30.8%), Bacteroidia (11.7-19.5%), and Alphaproteobacteria (3.7-8.6%). The hosts predicted for *Microviridae* matched the bacterial classes Negativicutes and Bacteroidia. Viral class Caudoviricetes had predicted hosts from all classes present except for Negativicutes and Nitrososphaeria. Unclassified viruses had hosts predicted within Bacteroidia, Alphaproteobacteria, Gammaproteobacteria, and Nitrososphaeria.

There were several classes that were predicted to be hosts but not observed within the 16S rRNA gene community. These were Negativicutes, Thermoproteia (Archaea), Paceibacteria, and Actinomycetia that were present in both Marker 28 and Riftia Field sites. Note that the 16S rRNA primers used do not target Archaea, which explains the absence of Thermoproteia in the amplicon data. Io did not have a 16S rRNA gene community analysis for comparison, however, it displayed similar predicted hosts to the other samples. While the classes Negativicutes, Thermoproteia, Paceibacteria, and Actinomycetia were not explicitly determined as part of the 16S rRNA gene community, the associated phyla Firmicutes, Thermoproteota, Patescibacteria, and Actinobacteriota were all present at or below 1% relative abundance (“Others”, S1 Fig and S1 Table).

## Discussion

### The viral community in microbial mats has the potential for influencing biogeochemical cycles

In this study, we sought to determine what viruses are present in iron-oxidizing microbial mat communities. These habitats are commonly found in hydrothermal vent fields at the base of sulfide chimneys and near/over diffuse hydrothermal vent flow [56]. Despite their high prevalence, their viral ecology has not previously been explored. We examined the natural viral community as well as the viral community after induction with mitomycin C. We found that, while 41% the viruses were shared between the three sites sampled here, there were differences in the relative abundances of vOTUs and AMG composition between the sites, and the AMGs detected were ecologically relevant for the iron-oxidizing mat community, which is exposed to reduced fluids and likely has organic carbon sticking to the iron-oxide matrix [20].

The AMGs abundant at each site highlight a variety of different metabolic pathways that indicate the potential to respond to different nutrient availability and environmental stresses on the microbial community. The viruses at the Io site displayed several mechanisms that capitalize on the use of sulfur in different ways, including the presence of the main metabolic pathway for assimilatory sulfate reduction, a pathway potentially providing electron donors for sulfate reducing bacteria [57], and the synthesis of cofactors that may enhance sulfur metabolism as well as other carbon and/or nitrogen metabolisms [58]. There was also the potential for viral manipulation of the cell envelope [59], which may increase cell membrane fluidity [60], consequently allowing easy passage of particular nutrients into the cell and fueling viral production. Further manipulation of host metabolism was possible with AMGs related to aldose scavenging [61] which could increase or supplement the carbon degradation repertoire of particular taxa, consequently manipulating carbon pools within the community. Additionally, AMGs related to a high-affinity complex for oxidative respiration [62–66] and heavy metal stress [67] were present in vOTUs at Io, indicating aerobic respiration could be maintained during infection, which could be advantageous during times when oxygen is limited. The host-virus interactions at the Io site suggests sulfur, low oxygen, heavy metals, and aldoses are ecologically important factors influencing to the microbial communities.

The AMGs unique to Marker 28 site demonstrated the potential for manipulation of a variety of nutrient cycles including carbon, sulfur, and nitrogen. Viruses at Marker 28 site encoded AMGs that participate in the tricarboxylate acid (TCA) cycle [68], autotrophic carbon fixation [69,70], and central carbon utilization [71], all of which could be involved in energy production for the virus [72]. Assimilatory sulfate reduction to incorporate sulfide into cysteine was targeted by two different AMGs, *cysH* and *cysC*, that could be targeting rate-limiting steps [73]. Additionally, there was an AMG for benzoate degradation that has been found as an electron donor for sulfate reducing bacteria (SRB), providing an additional carbon pathway [74]. Viruses targeted nitrogen and/or carbon cycling through an AMG unique to Marker 28 (*gdhA*), which could be used in a dissimilatory pathway to generate α-ketoglutarate to fuel the TCA cycle or assimilatory pathway that generates nitrogen [75,76], which could be used to generate more proteins, via more amino acid production, by phages for creation of progeny [77]. Tolerance to oxidative stress and maintenance of redox homeostasis may be attributed to the AMGs *gloB* and *gloC*, which participate in a methylglyoxal detoxification pathway [78,79]. These AMGs may be involved in niche creation for an otherwise anaerobic taxon. Marker 28 viruses may be targeting abundant taxa in a kill-the-winner strategy, where the most locally abundant taxa are infected and lysed more frequently [8,80]. This could have an impact on central carbon pathways and carbon fixation, as well as SRB by directly affecting the metabolic functioning of the microbial community. Viruses may also be providing novel functions to different taxa creating niches to reduce competition and streamline viral production. Overall, Marker 28 site viral assemblages highlight carbon fixation and utilization pathways, expanding or providing oxidative stress tolerance, as well as sulfur and nitrogen cycling as ecologically important processes within the microbial community.

Riftia Field viruses harbored strategies involving carbon cycling, potentially methane and sulfur as well as manipulating community dynamics. There were several AMGs related to the degradation of carbohydrates (*UGDH*), participation in the pentose phosphate pathway (*pgl*) [81], and cellulolytic capabilities (*cbhA*) [82,83], all of which could be ways viruses are acquiring energy for viral production. In a similar strategy, there was an AMG (*hprA*) suggested to be integral in formaldehyde oxidation or as part of the serine cycle for methylotrophic bacteria [84]. Supporting data was provided by the detection of methylotrophic order Methylococcales in the 16S rRNA analysis from the same samples. Additionally, there was an AMG (*glyA*), which may have a similar function to *hprA* of shunting carbon into the pentose phosphate pathway for either nucleotide synthesis and generating reducing power [85] or it may participate in methane metabolism by catalyzing an intermediate for methanogenesis. Assimilatory sulfate reduction AMGs (*cysH*) were present at Riftia Field similarly to the other two sites, but viruses at Riftia Field had a unique set of glycan biosynthesis AMGs. There was one set of AMGs (*rfbB/rffG*) involved in the production of vancomycin, streptomycin, and validomycin [86] and may either be imbuing resistance or a way to eliminate competition for resources [87]. Other glycan biosynthesis AMGs (*MGAT3, vanY*) may aid in biofilm formation through the creation of polysaccharides or increasing cell membrane fluidity [60] or can provide vancomycin resistance [88]. The combination of several AMGs related to remineralization of different carbohydrates as well as the presence of antimicrobials and resistance mechanisms may suggest that carbon is more limited at the Riftia Field site increasing the competition between the taxa [89]. Riftia Field is the only site where viruses have the potential ability to alter methane related metabolisms, which highlights this particular process as ecologically important at Riftia Field and but less so at the other sites.

Overall, the viral assemblages encoded genes involved in ecologically important functions within the microbial communities primarily related to carbon and sulfur rather than iron. While not statistically different, the virally-encoded AMGs were differentially distributed across sites, which is consistent with previous studies [25,90]. The temperatures between the sites were not drastically different, however, they do suggest different seawater and hydrothermal fluid interactions that could affect the geochemical gradients [91] producing a distinct microbial mat community and consequently viral assemblages in both composition and ecological functions within each site [90,92]. It is important to note that a large proportion of the community remained unclassified, with 29.1% − 99.6% vOTUs falling into “*unknown*” or “unclassified” consistent with other studies [90], which highlights the large and unexplored diversity within hydrothermal vents, but also makes it challenging to know the various hosts and biogeochemical cycles they impact. Additionally, the long storage time may have biased the surviving microbial community towards hardier taxa, which could decouple the induction results from the 16S rRNA amplicons for the host composition, i.e., the potential reason we did not see any Zetaproteobacteria viruses or even iron-related AMGs. Moreover, mitomycin C does not exist naturally in marine ecosystems [5], which may result in underestimating inducible lysogens by limiting burst size, i.e., number of phage progeny released, and limiting the growth of the phage in general [93]. Finally, due to the long storage time and short incubation period after mitomycin C treatment the number of inducible lysogens may have been limited.

Regardless, each site contains a distinct viral assemblage that performs different ecological roles and contains unique AMGs that highlight the different environmental factors that could be affecting the host community. The large proportion of shared vOTUs is likely the reflection of how similar these iron-oxidizing mats are as compared to other hydrothermal vent communities found in sediments, cold seeps, or plumes that share very little of their viral communities [90]. There is the possibility that the viruses may have different host ranges depending on which environment, i.e., host population, is present [94], adding nuance even to very similar communities. While the majority of the vOTUs were classified as unknown/unclassified, there are still important virus–host interactions that give insight into the role viruses play within iron-oxidizing mats. The viral assemblages have the potential to expand host carbohydrate usage, alter nutrient cycling through energy metabolism, and affect the surrounding community with antibiotic resistance or production and biofilm formation. The role of viruses within these communities is likely underestimated as only a small fraction of the proteins present within the vOTUs were annotated. Further exploration of virus–host dynamics within iron-oxidizing mats in hydrothermal vent systems like the EPR aid in constraining a vital component of the deep ocean food web.

### Host predictions highlight a broad range of affected taxa within iron oxidizing mats

Identifying the hosts of the vOTUs helps to contextualize the viral assemblages both in terms of which microbial members are present, and which potential metabolic functions are potentially being altered. If viruses are targeting active members of the community, determining which members and what potential functions they would be performing under infection would help to assess the impact these viruses have on the ecology of these iron-oxidizing mats. It may further reveal the unique structure of the different sampling sites and identify important virus–host interactions within the mat.

The identified host taxa represented a broad range of relative abundances from 50-55% (e.g., Classes Gammaproteobacteria, Bacteroidia, and Alphaproteobacteria), as well as less than 1% (e.g., phyla Firmicutes, Thermoproteota, Patescibacteria, and Actinobacteriota) suggesting the viruses can target both abundant and more rare taxa. The identification of an archaeal host may suggest that Archaea play an important role within iron-oxidizing mats albeit at low relative abundance as seen in other iron-oxidizing mats [19]. The low representation of archaeal ASVs within the 16S rRNA gene community reflects the use of bacterial-specific primers, which was done because they show less bias for these iron-oxidizing mats [95]. Gammaproteobacteria and Bacteroidia have been found to perform a variety of functions within iron-oxidizing microbial mats, including sulfur cycling, methanotrophy, aromatic carbon degradation, metal, and nitrogen cycling [96–100]. Viruses infecting these classes could have significant impacts to metabolic rates within the mat. The conspicuous absence of any Zetaproteobacteria hosts likely reflects both the novelty associated with the viral community with the majority only classified to the class or order level as well as a bias within the databases mined for host identification. Bias of the databases could also be the reason the predicted hosts were so similar across sites, and/or it could reflect how similar the microbial communities are themselves. Interestingly, lysogenic viruses were detected in Zetaproteobacteria-dominated iron-oxidizing mats at Kama’ehuakanaloa (formerly Loihi) Seamount by searching in MAGs [19]. In our work, only ~7.3% of the viral community had predicted hosts, which is consistent with other studies [90]. Combined, this indicates that much work is needed to improve host predictions for vOTU hosts in hydrothermal vent environments. Lack of knowledge about host specificity for vOTUs hampers our understanding of the potential impact of viruses within iron-oxidizing mats.

## Conclusion

Hydrothermal vents are found in every ocean basin and are important ecosystems to the overlying water column in terms of biogeochemical cycles, and serve as the base of the food web in the dark ocean. Viruses reflect the environmental conditions and physiological state of their hosts. Viruses are top-down controls that affect the diversity and abundance of the microbial community. Assessing rates of mortality, as well as improving host association with whole community metagenomics, is crucial to constrain the role and impact of viruses and rates of primary productivity. Here, we described the genetic novelty found in the viral communities sampled from iron-oxidizing microbial mats at 9˚50'N EPR. We showed that viral communities shared features but were specific to their site. Our work highlights important dynamics and host predictions to iron-oxidizing mats within the EPR and potentially other hydrothermal vent systems. Understanding the mechanisms that constrain and influence the viral communities has important implications to understanding and constraining microbial community processes and dynamics within hydrothermal vents that could affect primary productivity and consequently secondary production.

## Supporting information

S1 Fig Relative abundance of the 16S rRNA gene from Marker 28 and Riftia field sites. (TIFF)

S1 Table Amplicon sequence variant table of the 16S rRNA gene for Marker 28 and Riftia Field sites. (CSV)

S1 File S2-S5 Tables. Results from VIBRANT, checkV, genNomad, and iPHoP. Various tabular results from each of the softwares used to identify and classify vOTUs as well as predict hosts.
(XLSX)
